# Sun’s Rays in Consumption

**Published:** 1856-11

**Authors:** 


*Sun's Rays in Consumption.*

Dr. Coventry, in an Address before the New York State Medical Society, remarks:—"There is one subject which requires a more extended notice than it has usually received from our systematic writers. I refer to the influence of the sun's rays. Every physiologist knows how absolutely necessary they are to the growth of plants, and the etiolating effect their absence or withdrawal has upon the complexion. Is it unreasonable to suppose that they may have some influence in causing or preventing tuberculosis? It seems well established, that tubercles may be produced in animals by confining them in close and dark apartments, on a meagre diet. Doctor Hall says, that by this means he produced fatty degenerations in animals, which he considers analogous to, if not identical with, tuberculosis. In the city where I reside, there was an office, connected with a large mercantile establishment, so situated that the sun never shone upon it. It was in the rear of the building, with a single window, and that so surrounded with buildings as to exclude the sun. The occupants of the office died one after another, until the proprietors became alarmed, and had the office removed to another part of the building. One of the occupants I attended, when in the last stage of his disease. He entered the office a strong, healthy man, with no hereditary tendency to the disease, and temperate and regular in all his habits; but, in less than two years, he was carried, like his predecessors, to the grave, a victim to consumption. In his case I was never able to discover any cause, unless it was occupying that fatal office, where he was book-keeper."

